# Deep inferior epigastric perforator flap breast reconstruction in patients with Raynaud’s disease: a case series and literature review

**DOI:** 10.1093/jscr/rjaf535

**Published:** 2025-07-14

**Authors:** Rushabh Shah, Krzysztof Sosnowski, Amir Habeeb, Benjamin Smeeton, Charles M Malata

**Affiliations:** Department of Plastic and Reconstructive Surgery, Addenbrooke’s Hospital, Cambridge University Hospitals NHS Foundation Trust, Hills Rd, Cambridge CB2 0QQ, United Kingdom; School of Clinical Medicine, University of Cambridge, Hills Rd, Cambridge CB20SP, United Kingdom; Department of Plastic and Reconstructive Surgery, Addenbrooke’s Hospital, Cambridge University Hospitals NHS Foundation Trust, Hills Rd, Cambridge CB2 0QQ, United Kingdom; Department of Plastic and Reconstructive Surgery, Addenbrooke’s Hospital, Cambridge University Hospitals NHS Foundation Trust, Hills Rd, Cambridge CB2 0QQ, United Kingdom; Department of Plastic and Reconstructive Surgery, Addenbrooke’s Hospital, Cambridge University Hospitals NHS Foundation Trust, Hills Rd, Cambridge CB2 0QQ, United Kingdom; Cambridge Breast Unit, Addenbrooke’s Hospital, Cambridge University Hospitals NHS Foundation Trust, Hills Rd, Cambridge CB2 0QQ, United Kingdom; Anglia Ruskin University School of Medicine, Anglia Ruskin University, East Rd, Cambridge CB1 1PT, United Kingdom

**Keywords:** breast reconstruction, microvascular surgery, DIEP flap, Raynaud’s disease, vasospasm

## Abstract

Free tissue transfer using the deep inferior epigastric perforator (DIEP) flap represents the gold-standard for autologous breast reconstruction. Raynaud’s disease, characterized by vasospasm, thrombosis, and a hypercoagulable state, poses a theoretical risk to flap viability; however, its impact on DIEP outcomes remains poorly defined. We present the first reported case series evaluating DIEP flap reconstruction in three patients with Raynaud’s disease. In the first case, complete flap loss occurred due to thrombosis, in the absence of vasoprotective strategies. The subsequent two patients received intraoperative papaverine and verapamil to mitigate vasospasm, and postoperative antiplatelet therapy with aspirin and dipyridamole. Both achieved successful flap outcomes without microvascular complications. Our findings suggest Raynaud’s disease should not be considered a contraindication to DIEP flap reconstruction, provided tailored perioperative pharmacological strategies are employed. Larger studies are warranted to evaluate outcomes and guide evidence-based perioperative protocols in this understudied patient population.

## Introduction

The deep inferior epigastric artery perforator (DIEP) flap is widely regarded as the gold standard for post-mastectomy autologous breast reconstruction [1]. It involves transferring well vascularized abdominal soft tissue to recreate a soft durable, natural-appearing breast mound while preserving the rectus abdominis muscle. Compared with muscle-sacrificing or implant-based techniques, DIEP flaps offer reduced donor site morbidity, superior long-term aesthetic outcomes, and improved patient satisfaction [2]. However, free flap success hinges on reliable microvascular anastomoses, making patient-specific vascular risk factors highly relevant.

Raynaud’s disease is a vasospastic disorder characterized by transient digital ischaemia triggered by cold or emotional stress [3]. It can present idiopathically or as a manifestation of connective tissue disease, most commonly systemic sclerosis, where it is nearly universal [4]. The hallmark triphasic colour change (white–blue–red) reflects ischaemia, cyanosis, and reperfusion. Beyond episodic vasospasm, Raynaud’s has been associated with hypercoagulability, including impaired fibrinolysis and dense fibrin clot formation [3]. These features raise concern in the context of microvascular surgery, where both vasospasm and thrombosis can threaten flap perfusion.

Although hypercoagulability is recognized as a risk factor for microvascular compromise, the impact of Raynaud’s disease on free flap outcomes remains unclear [5]. To date, no case series has specifically examined its implications in free flap breast reconstruction.

We present, to our knowledge, the first case series evaluating DIEP flap reconstruction in patients with Raynaud’s disease. There was one flap failure and two successful reconstructions. We also explore the role of perioperative vasodilators and antiplatelet strategies in mitigating microvascular risk and improving flap outcomes.

## Case 1

A 53-year-old woman (Body Mass Index: 29.6) with a known history of Raynaud’s disease presented with right-sided multicentric breast cancer. Core biopsy confirmed invasive carcinoma with mixed ductal and lobular features, oestrogen receptor (ER) positive and HER2 negative. She was an ex-smoker, having stopped 25 years earlier, and was not on any regular medication for Raynaud’s.

**Figure 1:**
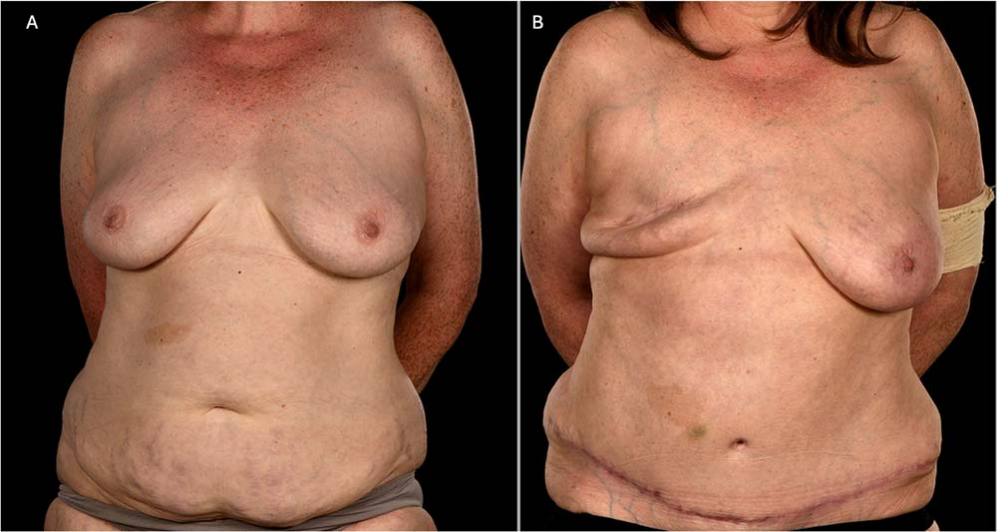
(A) Pre-operative photograph of patient 1 showing a smaller index right breast prior to mastectomy and adequate abdominal tissue for DIEP flap reconstruction. (B) Her 6-month post-operative shows a well-healed mastectomy scar following flap failure and primary closure. Please note the well-preserved inframammary fold.

She underwent a right-sided skin-sparing mastectomy (SSM) with sentinel lymph node biopsy, followed by immediate reconstruction with a DIEP flap. Preoperative CT angiography identified a single suitable medial row perforator (Fig. 1A).

Intraoperatively, the flap was harvested on one large superior periumbilical (medial row) and a small lateral row, central perforator supplied by an 8 cm vascular pedicle. A rib-sparing approach through the second intercostal space was used to access the internal mammary vessels [6]. Microvascular anastomoses were performed end-to-end using 9/0 nylon for the artery and a 3 mm venous coupler. Despite good immediate flow following release of vascular clamps, after several minutes, the flap rapidly became pale with fixed dermal staining. Suspecting vasospasm, likely due to the low ambient theatre temperature (21°C), topical lidocaine was applied, and 5000 IU of heparin with 10 ml of alteplase was administered via a small arterial side branch. This yielded transient clinical improvement.

Postoperatively, she received standard thromboprophylaxis with dalteparin 5000 IU once daily, and was nursed in a warm side room with hourly flap monitoring.

Within 6 hours, the Doppler signal over the pedicle was lost. Despite an initially reassuring clinical appearance, emergent re-exploration revealed complete arterial and venous thrombosis extending into the flap. Salvage was not possible, and the flap was excised. The mastectomy skin flaps were closed primarily.

The patient recovered uneventfully. At 6-month follow-up, she demonstrated a well-healed mastectomy scar with no signs of local complications (Fig. 1B). The flap failure was attributed to intra-flap thrombosis, likely exacerbated by untreated vasospasm and a hypercoagulable tendency associated with Raynaud’s disease. Three years later, she successfully underwent subpectoral expandable implant reconstruction.

## Case 2

**Figure 2:**
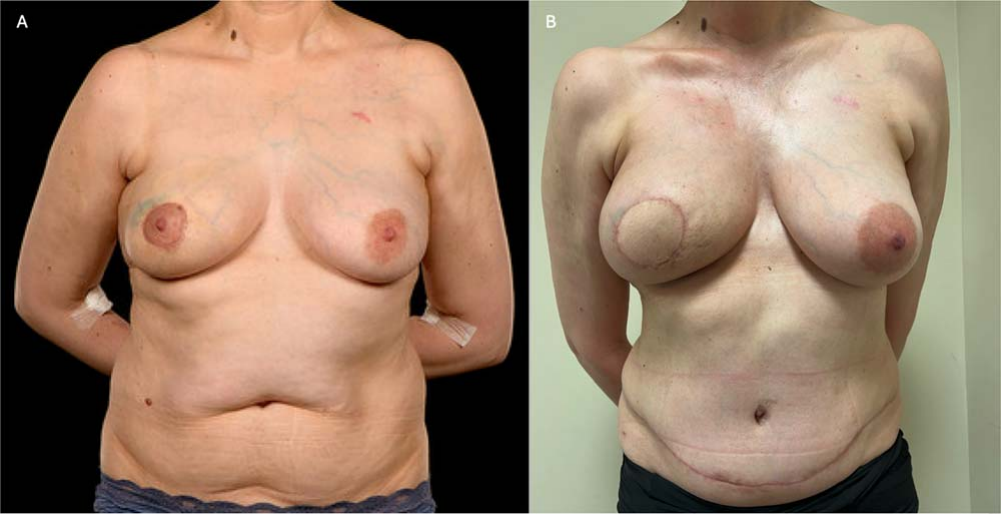
(A) Pre-operative photograph of patient 2 prior to right-sided skin-sparing mastectomy and DIEP flap reconstruction, showing the effects of periareolar lumpectomies. The margins were incomplete, necessitating a subsequent mastectomy. (B) Here she is shown 4 months post-operatively, and 3 weeks following completion of post-mastectomy radiotherapy. The flap has healed well with good volume and reasonable symmetry.

A 47-year-old woman (BMI 24.3) with a diagnosis of triple-negative invasive carcinoma of no special type underwent neoadjuvant chemotherapy, followed by wide local excision and sentinel lymph node biopsy. Residual invasive disease prompted a re-excision, but persistent tumour involvement led to a recommendation for completion mastectomy. She had a known history of Raynaud’s disease but was not taking any medication for it. Her preoperative appearance is shown in Fig. 2A.

She proceeded to a right-sided SSM with immediate DIEP flap reconstruction. The flap was raised on 2 large medial row perforators as predicted by preoperative CTA. It required a 6 cm intramuscular dissection. Intraoperatively, topical papaverine and verapamil were applied to the perforators and pedicle. A rib-sparing dissection of the second intercostal space exposed the internal mammary vessels, which were similarly treated with topical vasodilators. Two venous anastomoses were performed using a 3.5 and 2.5 mm couplers to the anterograde and retrograde internal mammary veins, respectively. The arterial end-to-end anastomosis was completed using interrupted 9/0 nylon sutures.

Postoperatively, the patient was nursed in a warm room, received intravenous fluids and oxygen therapy, and was administered standard thromboprophylaxis with dalteparin. She was also commenced on aspirin 75 mg once daily and dipyridamole 200 mg twice daily. She made a quick postoperative recovery and was discharged on Day 4. At 6-week follow-up, the flap demonstrated healthy perfusion, good volume, and satisfactory contour (Fig. 2B).

## Case 3

**Figure 3:**
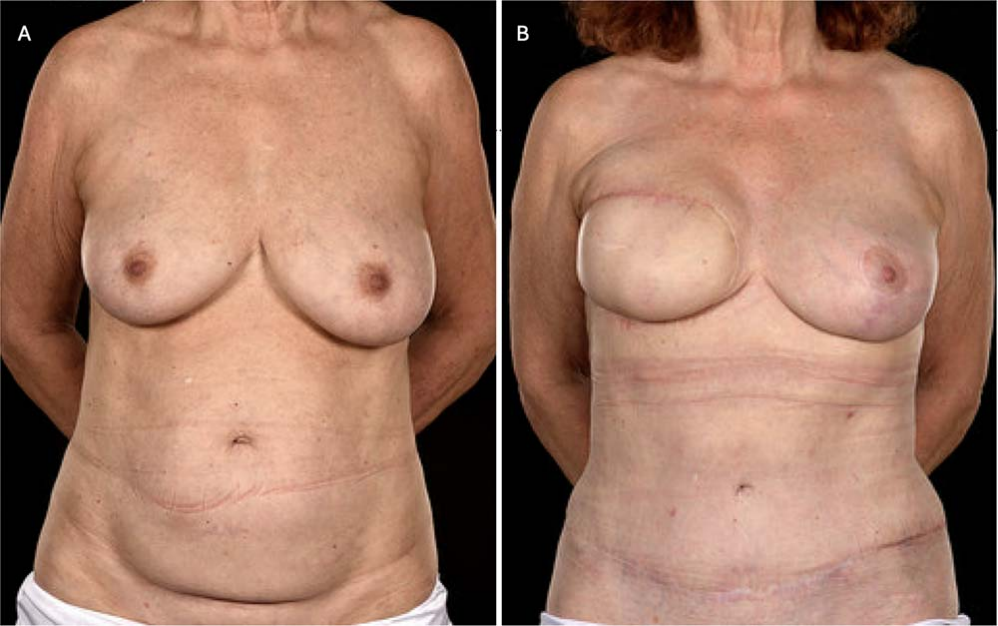
(A) Pre-operative photograph of patient 3 prior to bipedicled reconstruction for a right-sided breast cancer. The patient requested extensive skin resection (i.e. a simple mastectomy and axillary clearance), thereby necessitating wide cutaneous coverage which was to be achieved with a bipedicled DIEP flap in view of her thin body habitus and previous caesarean section scar. (B) Here she is shown 18 months postoperatively and12 months post-radiotherapy. She has reasonable symmetry with an improved abdominal appearance. Note that the contralateral breast was uplifted subsequent to the breast mound reconstruction.

A 60-year-old woman (BMI 23.6) with right-sided ER/PR-negative, HER2-positive invasive breast carcinoma was referred for a simple mastectomy and axillary clearance following neoadjuvant chemotherapy. Her prior sentinel lymph node biopsy had revealed metastatic disease. Her medical history included Raynaud’s disease, antiphospholipid syndrome, and psoriatic arthritis. Regular medications—Secukinumab and aspirin—were withheld preoperatively. Her preoperative appearance is shown in Fig. 3A.

Given the requirement for a large skin paddle and the presence of comorbidities, a bipedicled lower abdominal perforator flap reconstruction was planned. This comprised a right-sided DIEP flap (based on type I and II perforators) and a left-sided superficial inferior epigastric artery (SIEA) flap. Both pedicles were dissected to sufficient length, and all vessels-including the internal mammary artery and vein-were treated intraoperatively with topical papaverine and verapamil.

Rib-sparing internal mammary vessel exposure was performed through the second and third intercostal spaces. The right DIEP flap was anastomosed to the anterograde internal mammary vessels in the second space using a 3.0 mm versus coupler and 9/0 nylon for the artery. The left SIEA flap was anastomosed to the retrograde internal mammary vessels in the third space using a 2.5 mm coupler and 9/0 nylon. There was good immediate flow in both sets of anastomoses.

Postoperative care included nursing in a warm room, intravenous fluids, and standard thromboprophylaxis with dalteparin. The patient also received aspirin 75 mg once daily and dipyridamole 200 mg twice daily for 5 days. She was discharged on postoperative day 6 with an abdominal drain in situ. At 18-month follow-up, she demonstrated excellent breast symmetry and a well-healed abdominal donor site (Fig. 3B).

## Discussion

Raynaud’s disease is a well-recognized vascular disorder characterized by episodic vasospastic responses to cold and emotional stress. In reconstructive microsurgery, this presents a unique challenge. The disease's hallmark pathophysiology, which is marked by vasospasm, endothelial dysfunction, and a prothrombotic state, raises theoretical concerns about impaired perfusion within small vessels, hence increased risk of flap failure.

Vasospasm in Raynaud’s is mediated by increased sympathetic tone and elevated levels of vasoconstrictive mediators such as endothelin-1 [7]. In secondary forms associated with connective tissue disorders, chronic endothelial injury, intimal fibrosis, and circulating autoantibodies further exacerbate vascular instability [7, 8]. These processes contribute to a hypercoagulable state, increasing the risk of both arterial and venous thrombosis within microsurgical flaps. This was exemplified in Case 1, where an initially well-perfused flap developed extensive thrombosis and ultimately failed, despite standard thromboprophylaxis with dalteparin. Notably, no intraoperative vasodilators or postoperative antiplatelet agents were administered.

In contrast, Cases 2 and 3 received targeted perioperative pharmacological prophylaxis-topical intraoperative vasodilators (papaverine and verapamil) to mitigate vasospasm, and dual antiplatelet therapy (aspirin and dipyridamole) postoperatively (Table 1). Both cases achieved successful flap outcomes without microvascular complications. These observations support the hypothesis that modulation of vasomotor tone and platelet activity may enhance flap survival in patients with Raynaud’s disease.

**Table 1:** Summary of DIEP flap reconstructions in patients with Raynaud’s disease

**Feature**	**Case 1**	**Case 2**	**Case 3**
Age (years)	53	48	60
BMI (kg/m^2^)	29.6	24.3	23.6
Raynaud’s medication	None	None	Aspirin (stopped pre-op)
Other comorbidities	None	None	Antiphospholipid syndrome, psoriatic arthritis
Timing of reconstruction	Immediate	Immediate	Immediate
Flap type	Right DIEP (single pedicle)	Right DIEP (single pedicle)	Bipedicled (Right DIEP + Left SIEA)
Flap weight (grams)	737	623	584
Ischaemia time (mins)	126	101	139
Intraoperative vasodilators	Lidocaine only (rescue use)	Papaverine + Verapamil	Papaverine + Verapamil
Postoperative antiplatelets	None	Aspirin + Dipyridamole	Aspirin + Dipyridamole
Dalteparin	Yes (prophylaxis)	Yes (prophylaxis)	Yes (prophylaxis)
Flap Outcome	Complete flap loss	Successful	Successful

The use of vasodilators is well-established in microsurgical practice [9]. Papaverine, a phosphodiesterase inhibitor, and verapamil, a calcium channel blocker, act directly to relax vascular smooth muscle, hence reduce vasoconstriction [7]. Their application to recipient and donor vessels may attenuate the vasospastic component of Raynaud’s, as evidenced in our series. Chin *et al.* have similarly recommended vasodilator use and conservative flap design in patients with connective tissue disease, noting improved outcomes with subfascial dissection and judicious flap handling [8].

Postoperative antiplatelet therapy with aspirin and dipyridamole may further reduce the risk of microvascular thrombosis. Aspirin inhibits thromboxane A2-mediated platelet aggregation, while dipyridamole increases intraplatelet cyclic AMP levels, limiting activation and adhesion [10, 11]. Although data in the microsurgical setting are limited, Wang *et al*. reported favourable outcomes in hypercoagulable patients undergoing free tissue transfer with pharmacological prophylaxis [12]. In our series, the only flap failure occurred in the absence of antiplatelet prophylaxis, suggesting that dalteparin alone may be insufficient in this cohort.

Beyond pharmacology, other technical considerations are critical. Maintaining warm ambient temperature, avoiding excessive pedicle and perforator manipulation/traction, preserving multiple perforators where possible, and selecting appropriate flap dimensions help mitigate vasospasm and support optimal perfusion. Similarly, use of a free flap based on two vascular pedicles, as in patient 2 optimizes flap perfusion as it mimics the situation in vivo. In our cohort, all patients were nursed in side rooms with active warming, received intravenous hydration to ensure adequate perfusion, and underwent careful flap dissection and inset.

Current literature on microvascular reconstruction in patients with collagen vascular disorders is limited, with most studies focusing on general outcomes rather than disease-specific perioperative protocols. Wang *et al.* demonstrated successful free flap outcomes in hypercoagulable patients, including DIEP flaps, while Yan *et al.* reported low overall failure rates (3.8%–10.4%) in patients with collagen vascular disease [12–14]. However, none of these studies specifically addressed Raynaud’s disease or provided detailed perioperative strategies.

To our knowledge, this is the first case series describing outcomes of DIEP flap reconstruction exclusively in patients with Raynaud’s disease. While limited by sample size, our findings suggest that Raynaud’s is not an absolute contraindication to free flap surgery. Rather, it necessitates a heightened awareness of vasospastic and thrombotic risks and a tailored perioperative approach. The incorporation of intraoperative vasodilators and early postoperative antiplatelet therapy may be instrumental in improving outcomes as was the case in our second and third patients.

In conclusion, patients with Raynaud’s disease can safely undergo DIEP flap reconstruction when appropriate pharmacological and intraoperative strategies are implemented. Larger prospective studies and multicentre registries incorporating comorbidities are warranted to better define risk, refine perioperative protocols, and guide the development of formalized guidelines for this underserved patient population.
