# Correction to: Deep inferior epigastric perforator flap breast reconstruction in patients with Raynaud’s disease: a case series and literature review

**DOI:** 10.1093/jscr/rjag406

**Published:** 2026-05-19

This is a correction to: Rushabh Shah, Krzysztof Sosnowski, Amir Habeeb, Benjamin Smeeton, Charles M Malata, Deep inferior epigastric perforator flap breast reconstruction in patients with Raynaud’s disease: a case series and literature review, *Journal of Surgical Case Reports*, Volume 2025, Issue 7, July 2025, https://doi.org/10.1093/jscr/rjaf535

The following changes have been made to the originally published paper.

An **Author contributions** section has been added with the following statement: “Rushabh Shah and Krzysztof Sosnowski contributed equally to the manuscript.”

